# Plate Osteosynthesis or Figure-of-Eight Brace: Which One Is Better in Midshaft Clavicle Fractures?

**DOI:** 10.7759/cureus.14339

**Published:** 2021-04-07

**Authors:** Subodh Kumar Pathak, Rakesh K Gautam, Anil Godara, Manjeet Singh, Naveen Kumar, Aryan Sharma, Avin Vyas, Sameer Khan, Bijender Kumar, Mohammed Obair Mateen

**Affiliations:** 1 Orthopedics, Maharishi Markandeshwar (Deemed to be University), Ambala, IND

**Keywords:** clavicle, non union, functional score, dash score, ases score, figure-of-eight brace

## Abstract

Introduction

Fracture of the clavicle bone is a very common injury owing to its subcutaneous location. Controversy exists about the optimal treatment of midshaft clavicle fractures in the presence of significant displacement and comminution of the fracture. Traditionally, non-surgical management was considered the first treatment option for most clavicle fractures. However, recent evidence shows that the non-surgical option causes more complications than previously reported. The purpose of this study was to compare the clinical and radiological outcomes of conservative treatment and surgical treatment for midshaft clavicle fractures.

Materials and methods

A total of 45 patients meeting the inclusion criteria were included in this randomized study. The patients were allocated to two groups: conservative and operative on an alternate basis. Patients in the conservative group were managed with figure-of-eight bandage, whereas patients in the operative group were treated surgically by plate fixation. Primary outcome was recorded at six weeks, three months, six months, and 12 months follow-up using the Disabilities of the Arm, Shoulder, and Hand (DASH) and American Shoulder and Elbow Surgeons (ASES) scores. We also assessed patient’s satisfaction after the treatment, fracture union, and complication rates among the study cohort.

Results

The ASES scores were significantly better in the operative group at three months and six months follow-up; however, at 12 months follow-up, there was no significant difference in the score between the groups. Although not statistically significant, the DASH score was better in the operative group than in the conservative group at all the follow-ups. This study showed that the time to union was lesser, rate of non-union was lower, and return to work was faster on the operative group. The mean satisfaction score in the operative and conservative groups was 4.16±0.76 and 4.05±1.24, respectively (p = 0.76).

Conclusion

This study suggests that open reduction and internal fixation with plate reduced the incidence of mal-union and non-union; however, surgical treatment showed no significant difference in the functional outcome as compared to conservative treatment.

## Introduction

Fracture of the clavicle bone is a common injury encountered in emergency settings all over the world, accounting for nearly 2.6%-5% of all fractures and almost half of all shoulder girdle injuries [1]. The most common region being fractured is the middle third, accounting for almost 85% of all clavicular fractures [2-4]. Though ample amount of literature is available regarding the management of displaced midshaft clavicle fracture, no consensus has been made so far as to which treatment modality holds ground for optimally managing clavicle fracture. The archaic belief of orthopedic surgery training has nurtured such an approach of “benign neglect” for clavicle fracture management that even conservative treatment with sling or figure-of-eight bandage was criticized sometimes. Though conservative treatment is free of post-operative complications, it is associated with non-union, mal-union, pain, cosmetic deformity, and limitations of the shoulder movements, which cause significant functional disability [5-8]. The associated residual functional disability and clinical deformity after conservative treatment were readily accepted, whereas less emphasis was laid on the functional outcomes and patient satisfaction. Recently, many studies have shown that surgical treatment of displaced midshaft clavicle fractures in adults yields better clinical outcomes than conservative treatment [9-11]. However, a few studies have highlighted the ill effects of surgical intervention such as hardware failure, surgical wound infection, need for revision surgeries, and hypoesthesia [12-15]. Recently, a few meta-analyses comparing operative versus nonoperative approaches for the treatment of midshaft clavicle fractures have been published, with conflicting and inconclusive results [13,16,17]. The present study was designed to compare the effectiveness of conservative and operative methods for the treatment of displaced midshaft clavicle fractures in terms of functional outcome and complications.

## Materials and methods

This prospective randomized study was conducted at a tertiary care center after approval from the Institutional Ethical Committee. Fractures were classified according to Robinson’s classification. Patients in the conservative group were treated with figure-of-eight, where those in the operative group were treated with open reduction and internal fixation with plate. Patients with non-union, pathological fractures, open fractures, fractures with neurovascular injures, and clavicle fractures with major ipsilateral limb injury were excluded from the study.

Inclusion criteria

The inclusion criteria include the following: (1) age 18-60 years, (2) displaced middle third clavicle fractures (vertical displacement on anteroposterior view more than the width of the clavicle with no cortical contact), (3) Robinson’s classification types 2B1 and 2B2, (3) medically fit to undergo surgery (American Society of Anaesthesiologists [ASA] grade I, II, or III) and (4) willing to provide informed consent.

Patient cohort

Between 2018 and 2020, 45 patients with displaced midshaft clavicle fracture were included in the study and randomized to each treatment group on an alternate basis (the first patient was allocated to the operative group and then the next to conservative and so on alternatively). Furthermore, those who refused for surgery were also included in the conservative group. We assessed and compared the functional outcome and complications between these two treatment groups. Bone shortening was determined on an anteroposterior chest radiograph by measuring the distance between the sternal and acromial edges of each of the two clavicles. Shortening of more than 2 cm was considered significant. The fracture non-union was defined as a lack of radiographic healing after six months of treatment (surgical or conservative).

Outcome measures

Patients were examined clinically at six weeks, three months, six months, and 12 months, and Disabilities of the Arm, Shoulder, and Hand (DASH) and American Shoulder and Elbow Surgeons (ASES) scores were evaluated. Furthermore, shoulder radiographs were taken at each visit to assess the union status.

Primary functional outcome measures were (1) DASH and ASES scores and (2) fracture union time. Secondary outcome measures were (1) complications, (2) return to work, and (3) satisfaction with the treatment.

Satisfaction with the treatment was measured at 12 months follow-up by patient satisfaction score, where the patient was asked how satisfied he/she was after the treatment and the response were recorded in the format given in Table 1.

**Table 1:** Patient satisfaction score

Response	Score
Very satisfied	5
Somewhat satisfied	4
Neither satisfied nor dissatisfied	3
Somewhat dissatisfied	2
Very dissatisfied	1

Treatment modalities

Conservative Group

Immobilization was performed using a figure-of-eight brace (Tynor Orthotics, Punjab, India). Pendulum and Codman exercises were performed by patients for the initial three weeks followed by restricted active shoulder abduction and adduction exercises for another three weeks. At six weeks, full range of motion (ROM) exercises were started.

Operative Group

**Figure 1:**
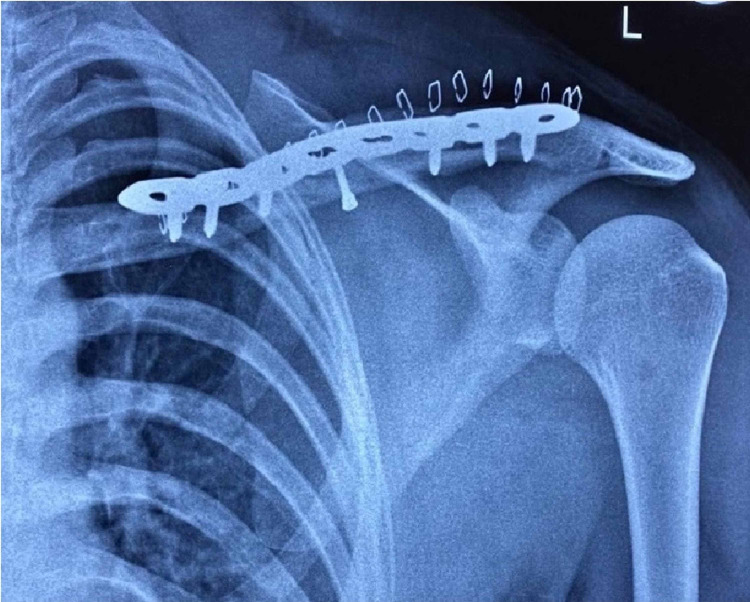
Post-operative radiograph showing the anatomical locking plate with screws in a clavicle fracture

All patients were operated within 10 days of injury. Patients were placed in the “beach-chair position”, with the entire extremity along with axilla prepared and draped. A linear incision was then made parallel to the clavicle shaft, and full-thickness skin and subcutaneous flaps were raised to expose the fractured ends. The fracture was reduced, and the clavicle was fixed with a titanium anatomical locking plate (Sharma Orthopedic (India) Private Limited, Vadodara, India) placed on the superior surface of the bone with at least six cortices in the medial fragment and six in the lateral fragment (Figure 1).

Immobilization with a sling was maintained for two weeks, until the skin sutures were removed. Post-operative rehabilitation protocol was similar to that of the conservative group.

Assessment of the Results

The DASH and the ASES scores at all times were calculated by two qualified physiotherapists who were not involved in the study. To ensure blinding, all the patients were instructed to wear the figure-of-eight brace during evaluation and not to reveal the treatment that they had undergone.

Statistical analysis

We used descriptive statistics to summarize demographics and outcome findings. Assessment of data distribution showed non-parametricity. We used Fisher’s exact test to compare categorical variables. The Mann-Whitney test and the repeated measures ANOVA (analysis of variance) test were used to compare continuous variables, as appropriate. Statistical significance was set at p < 0.05. All statistical analysis was performed using STATA v15.0 (StataCorp, College Station, TX, USA).

## Results

The final study group consisted of 42 patients, of which 18 patients underwent surgical intervention with plate (operative group) and 24 patients were managed conservatively (conservative group). One patient was lost at the six weeks follow-up and two others at three months follow-up and hence were excluded from the analysis. The mean age of the study group was 39.64 years (range: 18 to 57 years). There was no significant difference between the various groups in terms of distribution of age (p = 0.81), gender, and injured side (p = 0.46). The demographic data of patients is given in Table 2.

**Table 2:** Demographic details of the study cohort

Characteristics	Operative (n=18)	Conservative (n=24)	p-Value
Gender, n (%)			
Female	5 (27.7%)	7 (29.16%)	0.68
Male	13 (72.2%)	17 (70.83%)	0.85
Age, years (SD)	38.33 (8.52)	40.3 (9.75)	0.81
Dominant arm involvement (%)	11 (61.1%)	14 (58.3%)	0.46
Robinson type, n (%)			
2B1	12 (66.6%)	15 (62.5%)	0.35
2B2	6 (33.3%)	9 (37.5%)	0.42
Mode of injury, n			
Road traffic accident	15	21	0.716
Assault	1	3	0.437
Fall from a height	2	0	0.162

Primary outcome measures

DASH and ASES scores in both operative and conservative groups were assessed at six weeks, three months, six months, and 12 months. On analyzing the ASES scores at each follow-up, there was a statistically significant difference between the operative and conservative groups at three months and six months; however, there was no significant difference at 12 months follow-up (Table 3). The DASH score at all times was better in the operative group as compared to the conservative group; however, the difference was not statistically significant (Table 3).

**Table 3:** ASES and DASH scores at six weeks, three months, six months, and 12 months follow-up *Statistically significant values ASES, American Shoulder and Elbow Surgeons; DASH, Disabilities of the Arm, Shoulder, and Hand

ASES score	Operative group, mean (SD)	Conservative group, mean (SD)	p-Value
6 weeks	72.10 (6.69)	69.07 (5.69)	0.101
3 months	82.46 (6.27)	77.99 (5.19)	0.028*
6 months	90.01 (5.11)	85.86 (5.03)	0.022*
12 months	94.55 (5.97)	93.39 (4.10)	0.178
DASH score			
6 weeks	35.93 (4.59)	37.69 (4.89)	0.254
3 months	17.61 (3.54)	18.73 (4.53)	0.507
6 months	10.40 (3.04)	11.61 (3.78)	0.319
12 months	6.30 (2.64)	7.27 (2.92)	0.191

**Figure 2:**
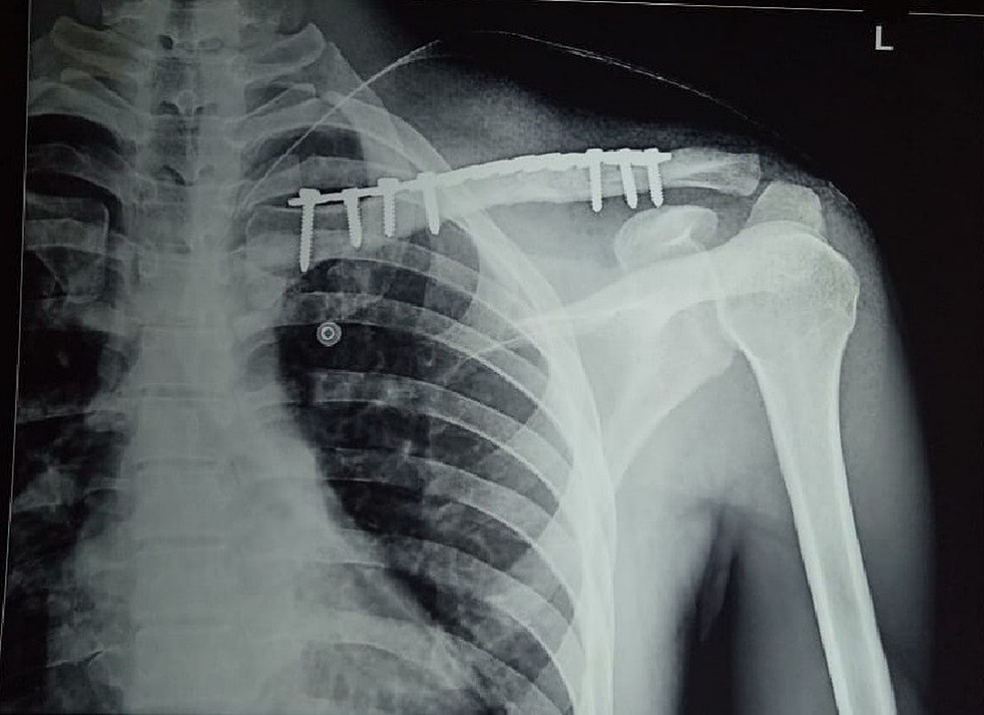
Radiograph of the left clavicle showing united fracture with locking plate in situ

**Figure 3:**
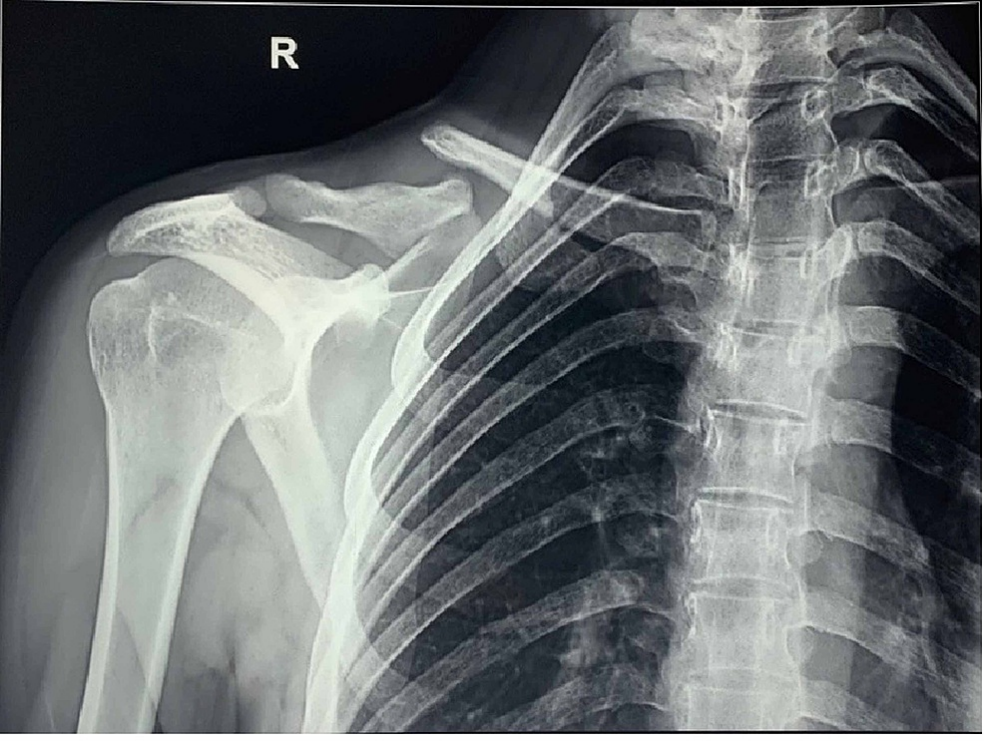
Radiograph of the conservatively treated right clavicle fracture showing non-union

**Figure 4:**
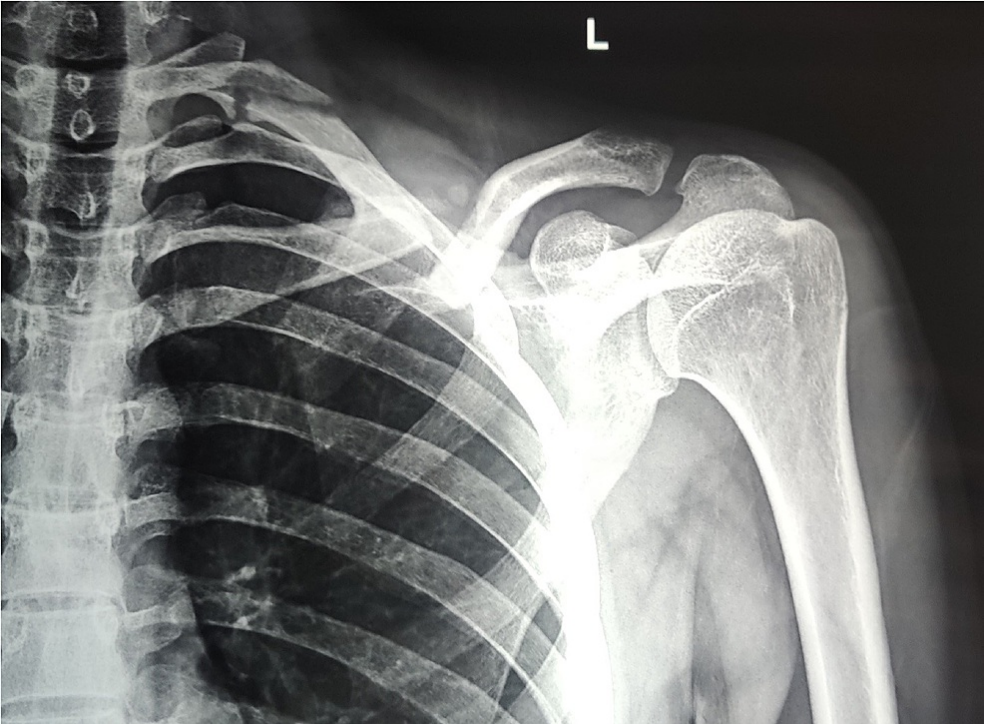
Radiograph showing left clavicle non-union in a patient managed conservatively

All fractures healed in the operative group (Figure 2), whereas two patients developed fracture non-union in the conservative group (p = 0.16) (Figures 3, 4).

The union rate in the operative group was 100% and that in the conservative group was 83.3%. Three patients in the operative group showed delayed union, with fracture uniting between four to six months. The mean union time was 14.21±2.03 weeks in the conservative group and 12.38±1.74 weeks in the operative group, which was statistically significant (p = 0.02).

Secondary outcome measures

There were a total of 15 complications in both groups (Table 4).

**Table 4:** Complications in the operative and conservative groups *Statistically significant values.

Complications	Operative group (%), (n=18)	Conservative group (%), (n=24)	p-Value
Non-union	0 (0)	2 (8.33)	0.16
Mal-union	0 (0)	3 (12.5)	0.08
Shortening (>2 cm)	0 (0)	2 (8.33)	0.16
Paresthesia	3 (16.6)	0 (0)	0.082
Hardware irritation	4 (22.2)	0 (0)	0.04*
Infection	1 (5.5)	0 (0)	0.33

**Figure 5:**
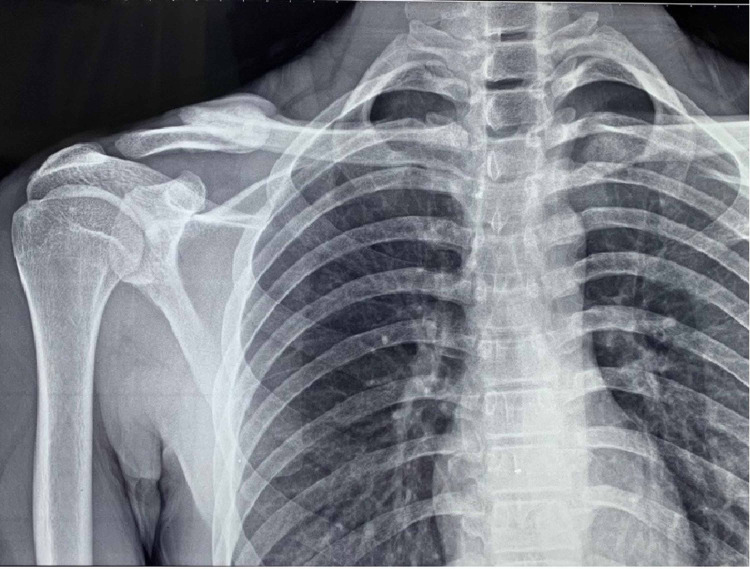
Radiograph showing a right clavicle mal-union with shortening at 12 months follow-up

The conservative group had two non-unions (8.33%) and three mal-unions (12.5%) (Figure 5), whereas the operative group had three patients with paraesthesia.

Four (22.2%) patients complained of plate irritation and prominence, of which one patient underwent revision surgery for implant removal at 11 months. One patient had superficial infection of the surgical site, which was treated with appropriate antibiotics. The average time of return to work in the operative group was 97±32.5 days and that in the conservative group was 123±41.5 days (p = 0.032). The mean satisfaction scores in the operative and conservative groups were 4.16±0.76 and 4.05±1.24, respectively (p = 0.76).

## Discussion

Managing displaced midshaft clavicle fractures with either surgical or conservative methods has always been controversial. As clavicle midshaft fracture occurs more commonly in the younger population, rapid recovery and early return to work, sports, or other activities are paramount. Therefore, primary fixation for midshaft clavicle fractures has gained momentum in the recent past, as evident by recent literature [18-22].

In our study, we have tried to make an ardent attempt to compare both of these treatment modalities in terms of their functional outcome as well as associated complications. In the study, we found no statistically significant differences between the two groups in terms of mode of injury, fracture type, patient demographics such as age/gender, and laterality. As our institution is in proximity to the state highway, we encounter a large amount of road traffic accidents (RTA), and midshaft clavicle fracture is commonly an associated injury among them. Hence, in our study, RTA was the most common mode of injury resulting in clavicle fractures. Furthermore, in our study, we found that males are more prone to sustain clavicle fracture as compared to females. This was in accord with the literature available [5,23].

The union rate in our study at the end of 12 months was 92.8% (n=39), and the mean time to union was 13.1 weeks (SD: 2.9). Union time in the operative group (12.38 weeks) was less as compared to the conservative group (14.28 weeks) (p = 0.02). Union rates of displaced midshaft clavicle fractures are reported to be between 90% and 100% when managed surgically with either plate or intramedullary fixation [15,24].

DASH score was slightly better in the operative group at all the follow-ups, but the difference was statistically insignificant. ASES score significantly improved in the operative group at three month and six months follow-up (p= 0.028, p= 0.02); however, the difference between the groups at the end of 12 months was statistically insignificant (p = 0.19). The literature review for functional outcome in terms of DASH score was inconclusive. Although a few meta-analyses have shown no difference in DASH scores between the operative and non-surgical treatment groups at one year after injury [11,25], a few other studies found higher DASH scores in the surgical treatment group than the non-surgical group [25,26].

The appearance of the shoulder imposes a significant factor on overall satisfaction of the procedure. Asymmetry of the shoulder can be associated with mal-union and non-union of the clavicle, whereas surgical scar over the neck region among the operative group is a matter of cosmetic concern especially among females. Patient satisfaction was greater in the operative group at the early follow-up times but approached near equal in the conservative group by 12 months. Five (20.8%) patients in the conservative group and four (26.6%) patients in the operative group had a patient satisfaction score of 3 or below. Dissatisfaction among the operative group was due to the presence of scar and plate prominence, whereas in the conservative group it was due to shortening, mal-union, or bony prominence. A similar finding was published by Tamaoki et al., who stated that surgery-caused scars led to the high dissatisfaction rate in the plate group. In their study of 98 patients with midshaft clavicular fractures, they found that 21.6 % patients in the surgical group were dissatisfied as compared to 14.9% in the non-surgical group [27].

We observed that conservative treatment resulted in more residual clavicle shortening than surgery, which was expected as surgical procedures are directed at achieving the best possible reduction. Prevalence of mal-union was as high as 12.5% in the conservatively treated group as compared to 0.0% in those treated operatively. In a study by Naveen et al. the rate of mal-union was as high as 20% in patients treated conservatively, whereas it was 3.3% in patients who underwent plate osteosynthesis [28]. Similarly, in a study of 30 patients, the authors reported mal-union as a complication in 43% of the patients treated conservatively and no case in patients managed with plate osteosynthesis [7]. We did not find any significant deficit in shoulder ROM of patients with clavicle shortening.

Given the subcutaneous location of the clavicle, the surgical treatment with plate can result in delayed wound healing, wound breakdown, or symptomatic hardware. The concept of “symptomatic hardware” was defined as prominent implant irritation or protrusions [4].In our study, 22.2% (n = 4) of patients had symptomatic hardware and one patient had implant removal at 11 months. Wang et al. found 40% of complications related to prominent hardware [4]. Dhakad et al. in their study of 50 patients reported prominent hardware in 8% of the patients [5].

In the literature, multiple studies report lower rates of non-union after plate fixation than conservative treatment, causing a shift toward surgical treatment [5,11,27,28]. In our study, the non-union rate in conservatively managed patients was 8.33% compared to no case of non-union in patients treated surgically. A few limitations of our study are poor randomization, uneven patient distribution among the groups, short follow-up period, and small sample size.

## Conclusions

In conclusion, this study did not demonstrate a difference in final functional outcomes between operative and conservative treatment of midshaft clavicle fractures; however, surgical treatment ensures faster recovery, early union, decreased likelihood of non-union, and early return to work or sports, and hence should be considered as and when patient profile and demand permit.
